# Supplementary material for: Probe Sequencing Analysis of Regenerating Lizard Tails Indicates Crosstalk Among Osteoclasts, Epidermal Cells, and Fibroblasts
Source: J Dev Biol. 2025 May 3;13(2):15. doi: 10.3390/jdb13020015 (PMC12101308; doi:10.3390/jdb13020015)
Supplement: Supplementary file 1 [file jdb-13-00015-s001.zip › jdb-3393047-supplementary.pdf]

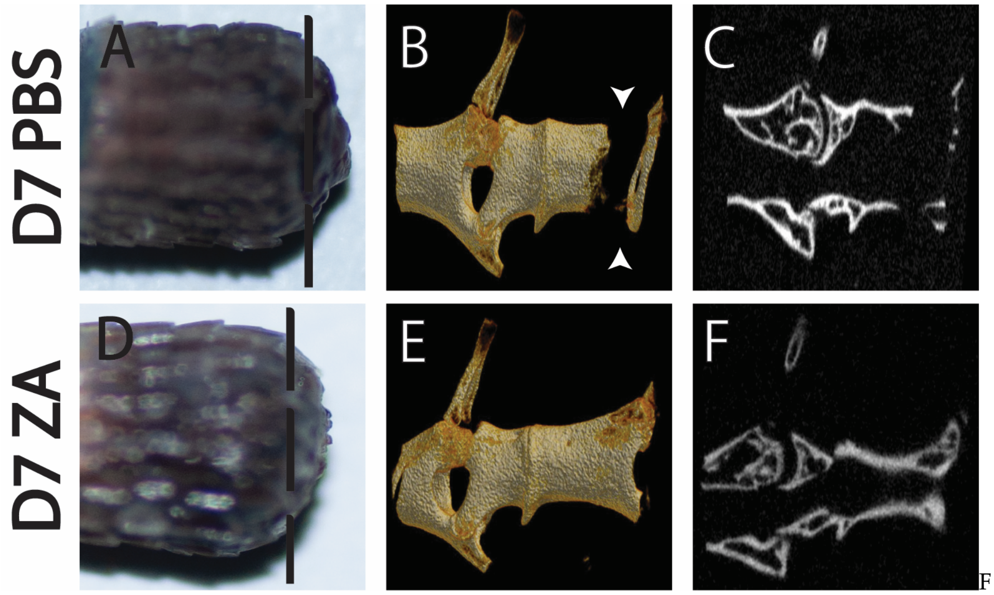

Supplemental Figure S1. Zoledronic acid (ZA) treatment prevents vertebral ablation and blastema formation. Tails were collected at D7 from **(A–C)** control lizards treated with PBS or **(D–F)** lizards treated with ZA and analyzed by **(A,D)** gross morphology, **(B,E)** u-CT 3D rendering, and **(C,F)** u-CT sagittal section.

Supplemental Table S1. PCR primers used for work with green anole (*Anolis Carolinensis*) cDNA.

Gene Name	Forward Primer	Reverse Primer
Gapdh	CCATGTTTGTGATGGGTGTC	CTTCTGTGTGGCTGTGATGG
Col1a1	CTGCTGGCAAGAATGGAGAC	GGACCTTGTTCACCAGGAGA
Spp1	AACCGGGAATCTGTCACCAT	GGCTATTCACATGCTCACGG
Hbd	TCAAGCTGCTGATCAAGTGC	CGAATGCTGGGAAGGGAAAA
Ifi30	AGTGCAGTTCCAGCCAGTAT	AAAATAGGCGCGACATGTCC
Col11a1	CTGTTGGCTTTCCTGGTGAC	TTTTCGCCTTGCCTTCCTTC
Col9a2	GTTAGCCGTGAGTGCCAAAA	CTCCCTGCACTCCTTTCTCA
Sox10	TGACAACCAGAGACATCCCC	CAGGGTGGAGATAGAGCAGG
Bcam	AAATGTTCCATTGCGCCACT	GCCAGGTCTCTTCCAACTCT
Myb	CACACAAGAGGCTGGGAAAC	CTGGAGTGTGAGCAAAACCC
